# Overweight and vitamin D deficiency are common in patients with irritable bowel syndrome - a cross-sectional study

**DOI:** 10.1186/s12876-024-03373-x

**Published:** 2024-09-03

**Authors:** Bodil Roth, Bodil Ohlsson

**Affiliations:** 1 Department of Internal Medicine, Skåne University Hospital, Jan Waldenströms Street 15, floor 5, Malmö, 205 02 Sweden; 2 Department of Clinical Sciences, Lund University, Malmö, Sweden

**Keywords:** Extraintestinal symptoms, Gastrointestinal symptoms, Irritable bowel syndrome (IBS), Micronutrients, Vitamin D, Weight

## Abstract

**Background:**

Irritable bowel syndrome (IBS) is a common disease with unknown etiology. Poor dietary intake with nutritional deficiency and overweight have been described to increase the risk of IBS. The aim of the present study was to compare weight and circulating levels of micronutrients in IBS compared with healthy controls.

**Design:**

Cross-sectional study.

**Methods:**

Patients diagnosed with IBS and healthy volunteers were recruited. Participants had to complete a dietary diary book and the questionnaires Rome IV, IBS-severity scoring system (IBS-SSS), and visual analog scale for IBS (VAS-IBS). Weight and height were measured, and blood samples were drawn. C-reactive protein (CRP), cobalamin, folate, iron, total iron-binding capacity (TIBC), and 25-hydroxy (25-OH) vitamin D were analyzed. Differences were calculated between groups and generalized linear model for regressions was adjusted for false discovery rate (FDR).

**Results:**

IBS patients (*n* = 260) were elder than controls (*n* = 50) (44.00 (33.25-56.00) vs. 37.85 (30.18–45.48) years; *p* = 0.012). After adjustment for age, both weight (β: 5.880; 95% CI: 1.433–10.327; *p* = 0.010, FDR = 0.020) and body mass index (BMI) (β: 2.02; 95% CI: 0.68–3.36; *p* = 0.003, FDR = 0.012) were higher in patients. Among IBS participants, 48.1% were overweight/obese compared with 26.0% in controls (*p* = 0.007). Diarrhea-predominated IBS had highest weight (*p* < 0.001) and BMI (*p* = 0.077). CRP and cobalamin were higher in patients than controls (*p* = 0.010 vs. *p* = 0.007), whereas folate was highest in controls (*p* = 0.001). IBS patients had lower intake of vegetables (*p* = 0.026), dairy products (*p* = 0.004), and cereals (*p* = 0.010) compared with controls. Despite 21.5% of IBS patients were taking vitamin D supplements, 23.65% of them had vitamin D levels below 50 nmol/L, compared with 26.0% observed in the control group (*p* = 0.720). Vitamin D levels were lower in overweight than in normal weight IBS patients (60 (48–73) nmol/L vs. 65 (53–78) nmol/L, *p* = 0.022). Vitamin D correlated with cobalamin and folate but correlated inversely with TIBC and BMI. IBS patients had a high degree of gastrointestinal and extraintestinal symptoms, which were inversely associated with iron levels. Extraintestinal symptoms were associated with increased BMI.

**Conclusion:**

IBS patients were often overweight or obese, with low vitamin D levels. High burden of extraintestinal symptoms were associated with overweight and lower iron levels.

**Registration:**

ClinicalTrials.gov, NCT05192603 (Date of registration 11/29/2021) and NCT03306381 (Date of registration 09/18/2017), respectively.

## Introduction

The etiology of irritable bowel syndrome (IBS) is unknown, but several factors such as female sex, dietary habits, psychological factors, gastrointestinal (GI) motility, visceral hypersensitivity, gut-brain axis dysfunction, low-grade intestinal inflammation, impaired epithelial barrier integrity, and gut microbiota alterations have been discussed as risk factors for the disease [1, 2]. Comorbidities with depression, anxiety, and pain from other organs are common in IBS [3–5]. Food intake is often a trigger to aggravated GI symptoms, and both a starch- and sucrose-reduced diet (SSRD) and a diet with low levels of fermentable oligosaccharides, disaccharides, monosaccharides, and polyols (FODMAP) have been shown to markedly improve GI symptoms, extraintestinal symptoms, and psychological well-being [6–9].

Patients with IBS/disorders of gut-brain interaction (DGBI) have been found to have a low intake and circulating levels of several micronutrients [6, 10]. This may be explained by poor food habits in IBS with high consumption of sugar and processed food [8, 11, 12]. A history of several restrictive diets, such as the low FODMAPs diet, to control abdominal pain and symptoms may lead to lower intakes of fruits, vegetables, and micronutrients [8, 9, 13]. Poor food habits for several years may lead to cognitive dysfunction, mental illness, and several organic conditions [14–16]. Both iron and vitamin D deficiency have become worldwide health issues [17, 18]. Iron deficiency and anemia are well-established causes of chronic fatigue [19]. Vitamin D deficiency may be involved in the development of central hypersensitivity and trigger both IBS symptoms and impaired psychological well-being [20–22], and is associated with many chronic diseases [23, 24]. Overweight and obesity are also growing health issues in the world, leading to several inflammatory and endocrine changes which can increase the risk for chronic diseases including IBS [25]. An association between vitamin D deficiency and overweight/obesity has been described [26, 27].

Our hypothesis was that overweight and low circulating levels of micronutrients may be of importance for experience of symptoms and psychological well-being in IBS. The aim of the present study was to examine weight and plasma/serum levels of micronutrients in IBS compared to healthy controls and whether these factors were associated with aggravated GI or extraintestinal symptoms and dietary habits.

## Materials and methods

### Patients

Patients with IBS were recruited to participate in a dietary intervention which took place at two different time points. The inclusion criteria for the dietary interventions were a diagnosis of IBS and age 18–70 years. Exclusion criteria were insufficient symptoms, i.e., < 175 scores on irritable bowel syndrome-severity scoring system (IBS-SSS) [28], alcohol or drug abuses, current eating disturbances, pregnancy, presence of any organic GI disease, severe GI surgery in the past, severe organic and psychiatric diseases, severe food allergy or on gluten-free-, vegan-, low FODMAP-, or low carbohydrate high fat (LCHF) diets.

**Fig. 1:**
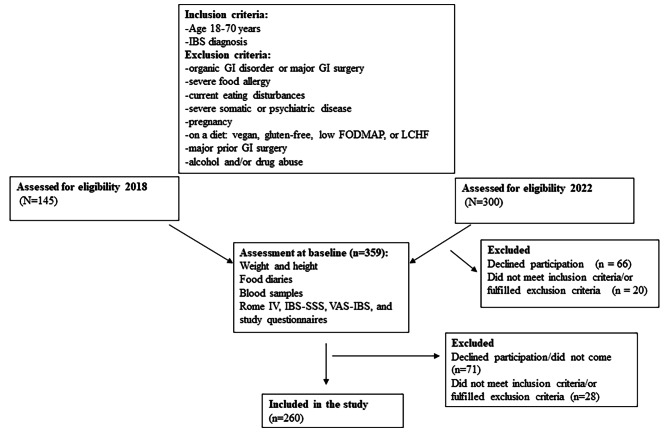
Flow chart over the recruitment process of patients with irritable bowel syndrome for randomization to either a diet with low content of fermentable oligo-, di- and monosaccharides and polyol (FODMAP) or starch- and sucrose-reduced diet (SSRD). LCHF = low carbohydrate high fat diet

The first cohort was included January 2018-February 2019, which is described in detail previously [6, 7]. Briefly, lists were provided over all subjects who had received an IBS diagnosis (K58.0 or K58.9 according to the International Statistical Classification of Diseases and Related Health Problems – ICD-10) in primary healthcare centers (PCC) or at the Department of Gastroenterology and Hepatology during 2015–2017. In total, 1,679 unique IBS patients were identified. Invitation letters were randomly sent to 679 patients, followed by a phone call a couple of weeks later. Initially, 145 patients were willing to participate, but 40 patients were later excluded because they did not show up, were not willing to participate at later time point, or did not fulfill the inclusion criteria. Thus, 105 patients (23 (21.9%) men) were finally included in the study, corresponding to 15% inclusion rate (Fig. 1).

The second recruitment process took place between December 2021 and September 2023 as described in detail [29]. Briefly, a data search was performed at Clinical Studies Sweden-Regional node for Southern Sweden from medical records of the County of Skåne for patients who had received an IBS diagnosis 2019–2022 according to the revised ICD-10 codes K58.1, K58.2, K58.3, and K58.9 [30]. Of the 3,587 patients in the area close to Malmö, 744 were randomly contacted by a written letter to inform about the study, providing all contact details to both investigators and encouraged to call or email the investigators whether they were interested of the study. Out of these, 58 were willing to participate in the study. One written referral was obtained from a private healthcare center and two phone call referrals from the Department of Gastroenterology were achieved. Eight patients contacted the investigators since they had been encouraged by the dietician or general practitioner (GP) at the PCC to participate. Nine patients contacted the investigators due to information leaflets in the waiting room at their PCC and four patients after recommendations from friends or relatives. Of these, 16 fulfilled the inclusion criteria. Two different campaigns in social media were performed by a professional company to recruit subjects with a diagnosis of IBS given by their physician (Trialy, Gothenburg, Sweden). The 218 patients who had assigned to participate in the study were contacted by phone by one of the investigators (BR), which led to that 140 patients were willing to participate and did not have any exclusion criteria. Out of all patients, some did not come to the first-time appointment (*n* = 53), did not fulfill the inclusion criteria of 175 scores in total IBS-SSS (*n* = 3), or fulfilled any exclusion criteria (*n* = 3) at the baseline assessment (Fig. 1). Finally, 155 IBS patients (72.4% of randomized cases; 48 (18.5%) men) could enter the dietary intervention (Fig. 1). This means an inclusion rate of 42.7% in the group recruited from social media and 6.5% in the group recruited from medical records.

#### Control subjects

Healthy controls were recruited among hospital staff and medical students at Skåne University Hospital, Malmö, through personal invitation and advertisement. The controls were not allowed to have any current chronic or acute illness or GI symptoms. Intake of multivitamins and hormonal contraceptive medicines was accepted, as well as temporary use of medications, such as seasonal allergy medicines and pain killers.

### Study design and clinical examination

Both patients and controls completed the study questionnaire with a diary book, and the questionnaires Rome IV, IBS-SSS, and visual analog scale for irritable bowel syndrome (VAS-IBS). All were examined for height and weight and body mass index (BMI) was calculated. BMI ≥ 25 kg/m^2^ was defined as overweight and BMI ≥ 30 kg/m^2^ was defined as obesity, according to the classification from the World Health Organization (WHO) [31]. Blood samples were drawn in a non-fasting state, and sent to the Department of Clinical Chemistry, Skåne University Hospital, for analyses.

### Questionnaires

#### Study questionnaire

All study participants were asked to complete a questionnaire regarding sociodemographic factors, smoking habits, weekly alcohol intake, minutes of physical activity per week leading to short of breath, pregnancies and childbirth, medical history, drug treatments, and family history. The IBS patients also had to answer questions about previous and current dietary modifications due to GI symptoms. All liquid and solid food intake was registered for 4 days (Wednesday-Saturday) [6].

#### Rome IV questionnaire

The Rome IV questionnaire was developed to diagnose DGBI [32]. Questions No 40–48 in the Swedish version of the questionnaire was used, after having received license from The Rome Foundation, Inc. Raleigh, NC, USA [33].

#### Irritable bowel syndrome-severity scoring system

IBS-SSS estimates abdominal pain, abdominal distension, satisfaction with bowel habits, and the impact of bowel habits on daily life using visual analog scales (VAS) ranging from absent (0 mm) to very severe (100 mm) symptoms. The number of days with abdominal pain in the last 10 days was reported. The maximum achievable score is 500. Scores ranging 75–174 indicate mild disease, 175–299 indicate moderate disease, and ≥ 300 indicate severe disease. Extraintestinal symptoms (nausea, difficulties to eat a whole meal, headache, back pain, fatigue, belching/excess wind, reflux, urinary urgency, leg pain, and muscle/joint pain) were estimated on VAS scales with maximal achievable score of 500 [28].

#### Visual analog scale for irritable bowel syndrome

The VAS-IBS covers the symptoms abdominal pain, diarrhea, constipation, bloating and flatulence, vomiting and nausea, psychological well-being, and intestinal symptoms’ influence on daily life, ranging from absent (0 mm) to very severe (100 mm) symptoms. The item psychological well-being has been shown to strongly correlate with anxiety in close relations, self-esteeism, and coping skills [34]. The values are inverted from the original format and validated to measure changes over time, with reference values for healthy controls [35, 36].

### Laboratory analyses

C-reactive protein (CRP), cobalamin, folate, iron, total iron-binding capacity (TIBC), and 25-hydroxy (25-OH) vitamin D were analyzed in plasma of patients and serum in controls according to clinical routines at the Department of Clinical Chemistry [37]. Reference values for healthy controls are available from the Department of Clinical Chemistry [37]. Due to changes in analysis routines at the laboratory, CRP, cobalamin, folate, and ferritin were only comparable between controls and the first IBS cohort.

### Statistical analyses

The statistical calculations were performed in IBM SPSS, version 29. Data were not normally distributed according to the Kolmogorov-Smirnov test and presented as median (interquartile ranges) or number (percentages). Age was categorized into age group < 29 years, 30–44 years, and > 45 years, according to the Public Health Agency of Sweden [38]. To assure that the two IBS cohorts could be merged, characteristics of the two cohorts were compared and calculated, before comparisons of the whole IBS cohort and controls. Mann-Whitney U-test, Kruskal-Wallis test, and Spearman’s test were used for continuous variables and Fisher’s exact test was used for dichotomous variables. Generalized linear model (GLM) was used to calculate differences between healthy volunteers and patients (predictor) with weight (adjusted for age) and laboratory analyses (adjusted for age and weight) as dependent variables, and to calculate differences in IBS patients regarding weight, BMI, and circulating levels of CRP and micronutrients (predictors) with GI and extraintestinal symptoms as dependent variables, adjusted for age and weight (laboratory analyses). Values are given as β and 95% confidence interval (CI). To adjust for multiple comparisons in the GLM calculations, crude *p*-values as well as the *p*-values adjusted for false discovery rate (FDR) set at 5% according to the Benjamin-Hochberg method were performed [39]. The FDR-adjusted *p*-values were the main results. *P* < 0.05 was considered statistically significant.

## Results

### Basal characteristics

Altogether, 260 IBS patients were included who fulfilled the Rome IV criteria [1]. Of these, 90 had mixed IBS (IBS-M), 70 had diarrhea-predominated IBS (IBS-D), 46 had constipation-predominated IBS (IBS-C), and 10 had unspecified IBS (IBS-U). Forty-one had unspecified functional bowel disorder (FBD), since they suffered from abdominal pain at least weekly, but without clear association between pain and altered bowel habits. Three patients had not completed the Rome IV questionnaire. The prevalence of IBS subgroups differed between sexes, with most men having IBS-D (40.0%) and most women having IBS-M (37.7%) (*p* = 0.040).

The age (*p* = 0.094), sex distribution (*p* = 0.257), weight (*p* = 0.862), education (*p* = 0.488), smoking habits (*p* = 0.984), alcohol intake (*p* = 0.661), or physical activity (*p* = 0.636) were equal between the two IBS cohorts. In the latter cohort, more were married/living together (*p* < 0.001) and working full time or being students with fewer retirements (*p* = 0.036) (Table 1). There were no differences regarding the severity of specific GI symptoms in VAS-IBS or total extraintestinal IBS-SSS, and the total IBS-SSS were equal in the first and second cohort (310 (248–353) vs. 300 (238–352), *p* = 0.352).

**Table 1:** Basal characteristics

Parameters	Controls *N* = 50	IBS 2018 *N* = 105	IBS 2022 *N* = 155	IBS patients *N* = 260	*P*-value*	*P*-value**
**Age (year)**	37.85 (30.18–45.48)	46.00 (34.50–57.00)	42.00 (32.00–55.00)	44.00 (33.25-56.00)	0.094	0.012
**Gender (male/female) (n**,**%)**	13 (26)/37 (74)	23 (22)/82(78)	25 (16)/130(84)	48 (18.5)/212 (81.5)	0.257	0.244
**Weight (kg)** *Missing*	64.6 (56.8–75.9)	71.4 (63.0-82.5) 6	69.2 (63.0-82.9)	70.5 (63.0-82.8) 6	0.862	0.002
**BMI (kg/m** ^**2**^ **)** *Missing*	22.6 (20.8–25.2)	24.3 (22.3–28.0) 7	25.0 (22.6–28.2) 1	24.8 (22.4–27.8) 8	0.854	< 0.001
**Education (n**,**%)**					0.488	0.017
*Missing*		2	1	3		
Primary school		5 (4.8)	7 (4.5)	12 (4.6)		
Secondary school	3 (6.0)	23 (21.9)	23 (14.8)	46 (17.7)		
Education after secondary school	9 (18.0)	23 (21.9)	37 (23.9)	60 (23.1)		
Examination at university	38 (76.0)	52 (49.5)	87 (56.1)	139 (53.5)		
**Occupation (n**,**%)**					0.036	0.038
*Missing*		3	1	4		
Working full time	35 (70.0)	53 (50.5)	93 (60.0)	146 (56.2)		
Working 99 − 51%	8 (16.0)	13 (12.4)	15 (9.7)	28 (10.8)		
Working 50%		6 (5.7)	2 (1.3)	8 (3.1)		
Studying	7 (14.0)	5 (4.8)	20 (12.9)	25 (9.6)		
Sick leave		5 (4.8)	5 (3.2)	10 (3.8)		
Unemployment		3 (2.9)	5 (3.2)	8 (3.1)		
Retirement		16 (15.2)	14 (9.0)	30 (11.5)		
Other		1 (1.0)	0	1 (0.4)		
**Marital status (n**,**%)**					< 0.001	0.237
*Missing*		2	1	3		
Living alone	13 (26.0)	41 (39.0)	30 (19.4)	71 (27.6)		
Living together	37 (74.0)	62 (59.0)	110 (71.0)	172 (66.2)		
Other				17 (6.5)		
**Smoking (n**,**%)**					0.984	0.091
Never	36 (72.0)	54 (51.4)	84 (54.2)	138 (53.1)		
Former	10 (20.0)	37 (35.2)	54 (34.8)	91 (35.0)		
Present un regular	1 (2.0)	5 (4.8)	8 (5.2)	13 (5.0)		
Present regular	3 (6.0)	6 (5.7)	8 (5.2)	14 (5.4)		
**Alcohol intake for 1 week (standard glass) (n**,**%)**					0.661	0.192
*Missing*		2	1	3		
< 1	17 (34.0)	46 (43.8)	67 (43.2)	113 (43.5)		
1–4	28 (56.0)	40 (38.1)	57 (36.8)	97 (37.3)		
5–9	4 (8.0)	12 (11.4)	26 (16.8)	38 (14.6)		
10–14	1 (2.0)	3 (2.9)	3 (1.9)	6 (2.3)		
≥ 15		2 (1.9)	1 (0.6)	3 (1.2)		
**Physical activity for 1 week (n**,**%)**					0.636	0.251
*Missing*		2	1	3		
No time	3 (6.0)	11 (10.5)	18 (11.6)	29 (11.2)		
< 30 min	4 (8.0)	24 (22.9)	25 (16.1)	49 (18.8)		
30–60 min	13 (26.0)	16 (15.2)	30 (19.4)	46 (17.7)		
60–90 min	7 (14.0)	12 (11.4)	24 (15.5)	36 (13.8)		
90–120 min	6 (11.8)	14 (13.3)	16 (10.3)	30 (11.5)		
< 120 min	17 (34.0)	26 (24.8)	41 (26.5)	67 (25.8)		

Physical activity means activity that leads to short of breath. Mann-Whitney U-test for comparisons between the two IBS cohorts* and between controls and the whole IBS cohort**. Values are given as number and percentages and median and interquartile range. *P* < 0.05 was considered statistically significant

Controls had more education at university level and were more often working full time with no sick leave and no retirements (Table 1). The patients had severe GI symptoms (Table 2), whereas the controls had low degree of symptoms within the reference values for healthy volunteers [36]. The highest scored item on VAS-IBS was psychological well-being which was graded to 5 (0–22) mm.

**Table 2:** Gastrointestinal symptoms

	IBS patients *N* = 260
**VAS-IBS (mm)**	
Abdominal pain 5 (1–13)	50 (33–65)
Diarrhea 3 (0–10)	51 (10–74)
Constipation 6 (2–16)	50 (4–73)
Bloating and flatulence 10 (2–23)	75 (58–87)
Vomiting and nausea 2 (0–4)	13 (2–38)
Intestinal symptom´s influence on daily life 2 (0–14)	71 (54–83)
Psychological well-being 5 (2–15)	46 (18–64)
**IBS-SSS**	
Total IBS-SSS	306 (242–353)
Total extraintestinal IBS-SSS	174 (119–244)
Nausea	13 (2–36)
Difficulties to eat a whole meal	10 (1–26)
Headache	28 (9–62)
Back pain	31 (6–65)
Fatigue	65 (36–84)
Belching/excess wind	71 (48–86)
Reflux	19 (4–55)
Urinary urgency	24 (4–68)
Leg pain	2 (0–16)
Muscle/joint pain	30 (6–67)

Specific gastrointestinal symptoms were measured by the visual analog scale for irritable bowel syndrome (VAS-IBS) [35]. Reference values of healthy volunteers for visual analog scale for irritable bowel syndrome (VAS-IBS) given in brackets [36]. Irritable bowel syndrome-severity scoring system (IBS-SSS). > 175 is defined as moderate IBS, which was the inclusion criteria for the study [28]. Median and interquartile range

The most common comorbidities in IBS were allergy (13.1%), eczema (9.2%), and reflux/hiatus hernia (8.8%). Eating disturbances was not asked for in the first cohort, but 18.1% of the IBS patients from the latter cohort had a history of any eating disturbances. No one had any actual eating disturbances. The most often used drugs were paracetamol (38.1%), non-steroid anti-inflammatory drugs (NSAID) (35.0%), and proton pump inhibitors (PPI) (24.6%). IBS patients used several supplements such as minerals (13.5%), multivitamins (11.5%), and vitamin B/folic acid (8.8%). As much as 21.5% of the IBS participants used any kind of vitamin D supplements, including pure vitamin D supplements (15.4%). Also, probiotics were frequently used (13.1%) (Table 3).

**Table 3:** Comorbidity and drug use in controls and irritable bowel syndrome

Comorbidity	Controls *N* = 50	IBS 2018 *N* = 105	IBS 2022 *N* = 155	IBS *N* = 260	*P*-value
Allergy	4 (8.0)	17 (16.2)	17 (11.0)	34 (13.1)	0.262
Anxiety	0	3 (2.9)	7 (4.5)	10 (3.8)	0.744
Asthma bronchialis	2 (4.0)	11 (10.5)	7 (4.5)	18 (6.9)	0.081
Burned out	0	5 (4.8)	10 (6.5)	15 (5.8)	0.787
Depression	0	11 (10.5)	10 (6.5)	21 (8.1)	0.255
Eczema	3 (6.0)	5 (4.8)	19 (12.3)	24 (9.2)	0.049
Fibromyalgia	0	3 (2.9)	9 (5.8)	12 (4.6)	0.371
Hypertension	0	10 (9.5)	12 (7.7)	22 (8.5)	0.654
Hypothyroid disease	0	12 (11.4)	6 (3.9)	18 (6.9)	0.024
Lactose intolerance	0	5 (4.8)	10 (6.5)	15 (5.8)	0.787
Migraine/headache	1 (2.0)	7 (6.7)	10 (6.5)	17 (6.5)	1.00
Reflux/hiatushernia	0	5 (4.8)	18 (11.6)	23 (8.8)	0.074
**Drug treatment**					
Allergy medicines	2 (4.0)	11 (10.5)	24 (15.5)	35 (13.5)	0.272
Antidepressants	2 (4.0)	18 (17.1)	23 (14.8)	41 (15.8)	0.609
Asthma inhalators	0	6 (5.7)	8 (5.2)	14 (5.4)	1.00
Hormonal treatment*	4 (8.0)	8 (7.6)	24 (15.5)	32 (12.3)	0.082
Laxatives/bulking agents	0	14 (13.3)	38 (24.5)	52 (20.0)	0.028
Levaxine	0	13 (12.4)	6 (3.9)	19 (7.3)	0.014
NSAID	1 (2.0)	41 (39.0)	50 (32.3)	91 (35.0)	0.290
Paracetamols	1 (2.0)	45 (42.9)	54 (34.8)	99 (38.1)	0.196
Proton pump inhibitors	1 (2.0)	16 (15.2)	48 (31.0)	64 (24.6)	0.005
**Supplements**					
Iron	0	1 (1.0)	7 (4.5)	8 (3.1)	0.148
Other minerals (Ca, Mg, Z, Si)	0	9 (8.6)	26 (16.8)	35 (13.5)	0.065
Vitamin B/Folic acid	0	9 (8.6)	14 (9.0)	23 (8.8)	1.00
Vitamin D	0	14 (13.3)	26 (16.8)	40 (15.4)	0.448
Multivitamins	1 (2.0)	7 (6.7)	23 (14.8)	30 (11.5)	0.049
Vitamin D and/or multivitamins	1 (2.0)	16 (15.2)	39 (25.2)	56 (21.5)	0.064
Probiotics	0	19 (18.1)	15 (9.7)	34 (13.1)	0.061

*= combination pills. Values are given as number (percentage). Differences were calculated between the two IBS cohorts by Fisher´s exact test. *P* < 0.05 was considered statistically significant

Among controls, allergy (8.0%), eczema (6.0%), and exercise-induced asthma (4.0%) were found, and the most frequently used drug was anti-conception combination pills (8.0%), with no use of vitamin or mineral supplement (Table 3).

### Weight and BMI

**Fig. 2:**
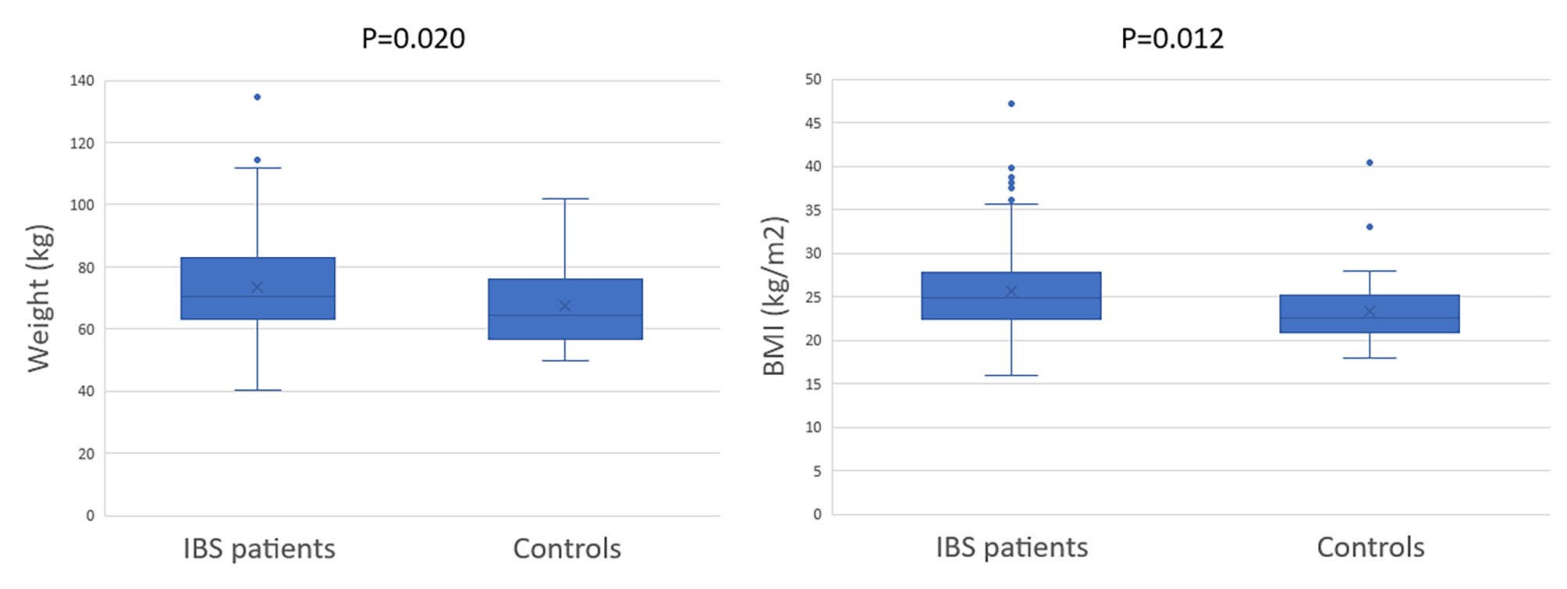
Differences in weight and body mass index (BMI) between IBS patients and healthy controls after adjustment for age in generalized linear model and p-values adjusted for false discovery rate (FDR). *P* < 0.05 was considered statistically significant

**Fig. 3:**
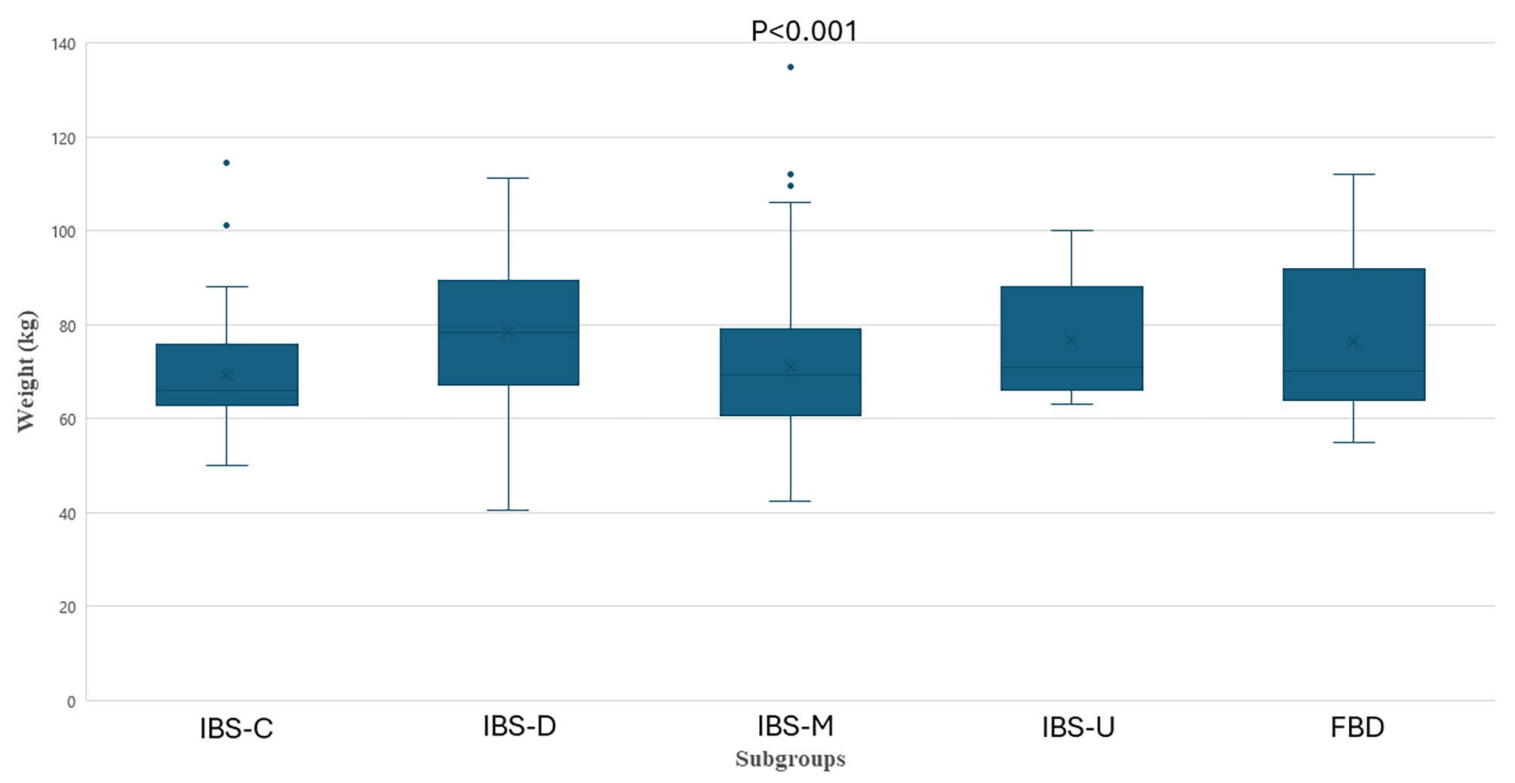
Differences in weight depending on subgroup of irritable bowel syndrome (IBS)/disorder of gut-brain interaction (DGBI). IBS-C = constipation-predominated IBS (*n* = 46), IBS-D = diarrhea-predominated IBS (*n* = 70), IBS-M = mixed IBS (*n* = 90), IBS-U = unspecified IBS (*n* = 10), FBD = functional bowel disorder (*n* = 41). Kruskal Wallis test. *P* < 0.05 was considered statistically significant

The patients with IBS were elder than controls (44.00 (33.25-56.00) years vs. 37.85 (30.18–45.48) years, *p* = 0.012) and weighed more with higher BMI (Table 1). The difference remained after adjustment for age regarding weight (β: 5.880; 95% CI: 1.433–10.327; *p* = 0.010, FDR = 0.020), as well as for BMI (β: 2.02; 95% CI: 0.68–3.36; *p* = 0.003, FDR = 0.012) (Fig. 2). Among IBS participants, 127 patients (48.8%) were normal weight, 90 (34.6%) were overweight, and 35 (13.5%) were obese, whereas among controls, 37 subjects (74.0%) were normal weight, 11 (22.0%) overweight and 2 (4.0%) obese (*p* = 0.007). When stratified into age groups, IBS patients had higher BMI than controls in age 18–29 years (*p* = 0.021), with overweight/obesity in 47.8% in IBS compared with 9.1% in controls (*p* = 0.037). In age 30–44 years, corresponding figures were 42.4% vs. 26.9%, *p* = 0.176, and in the age above 45 years the prevalence was 55.4% vs. 38.5%, *p* = 0.261. Weight (*p* < 0.001) and BMI (*p* = 0.077) differed among IBS subgroups, with highest weight and BMI in patients with IBS-D (78.3 (67.0-89.6) kg and 26.2 (22.7–29.6) kg/m^2^, respectively) and lowest in IBS-C (66.0 (62.8–75.8) kg and 24.1 (22.2–26.2) kg/m^2^, respectively) (Fig. 3). In a sensitivity analysis including only women, the weight was still highest in IBS-D (72.0 (64.4–86.2) kg, *p* = 0.019). There were no differences in weight depending on any current diets (data not shown).

### Laboratory analyses

More patients (18.1%) than controls (6.0%) had CRP levels above reference values (*p* = 0.049). IBS patients had higher cobalamin levels and lower folate levels than controls, although all controls were within reference values in contrast to IBS patients (Table 4). After adjustment for age and weight, levels of ferritin (β: 31.498; 95%: -2.559-65.554, *p* = 0.070, FDR = 0.070) and TIBC levels (β: -3.254; 95%: -6.447-(-0.061), *p* = 0.046, FDR = 0.061) were equal between patients and controls.

**Table 4:** Laboratory analyses in controls and irritable bowel syndrome

	Controls *N* = 50	IBS 2018 *N* = 105	IBS *N* = 260	*P*-value*	*P*-value**
**CRP (< 3 mg/L)** Above (n (%))	0.66 (0.35–1.35 3 (6.0)	0.72 (0.60–1.72) 19 (18.1)		0.010	0.049
**Cobalamin** **(150–500 pmol/L)** Below (n (%))	296 (235–366) 0	364 (259–429) 2 (1.9)		0.007	1.00
**Folate** **(≥ 6 nmol/L)** Below (n (%))	19 (15–24) 0	14 (10–21) 4 (3.8)		0.001	0.306
**Ferritin** **(13–148 µg/L)** Below (n (%))	36 (15–74) 9 (18.0)	69 (42–139) 8 (7.6)		< 0.001	0.022
**Iron** **(9–34 µmol/L)** Below (n (%))	16 (12–21) 3 (6.0)		18 (14–22) 13 (5.0)	0.178	0.731
**TIBC** **(47–80 µmol/L)** Above (n (%))	67 (61–72) 8 (16.0)		62 (56–69) 18 (6.9)	0.002	0.094
**Transferrin saturation (%)**	26 (20–36)		26 (19–34)	0.874	
**25-OH Vitamin D (> 75 nmol/L)** Below (n (%))	63 (49–78) 37 (74.0)		62 (50–74) 200 (76.9)	0.826	0.714

CRP = C-reactive protein, TIBC = total iron-binding capacity, 25-OH = 25-hydroxy. Serum was analyzed in controls and plasma in patients, which is equivalent with the same reference values (within brackets) according to the Department of Clinical Chemistry [37]. Mann-Whitney U test* and Fishers exact test** for the number below or above reference values. Values are given as median and interquartile range and number and percentages. *P* < 0.05 was considered statistically significant

**Fig. 4:**
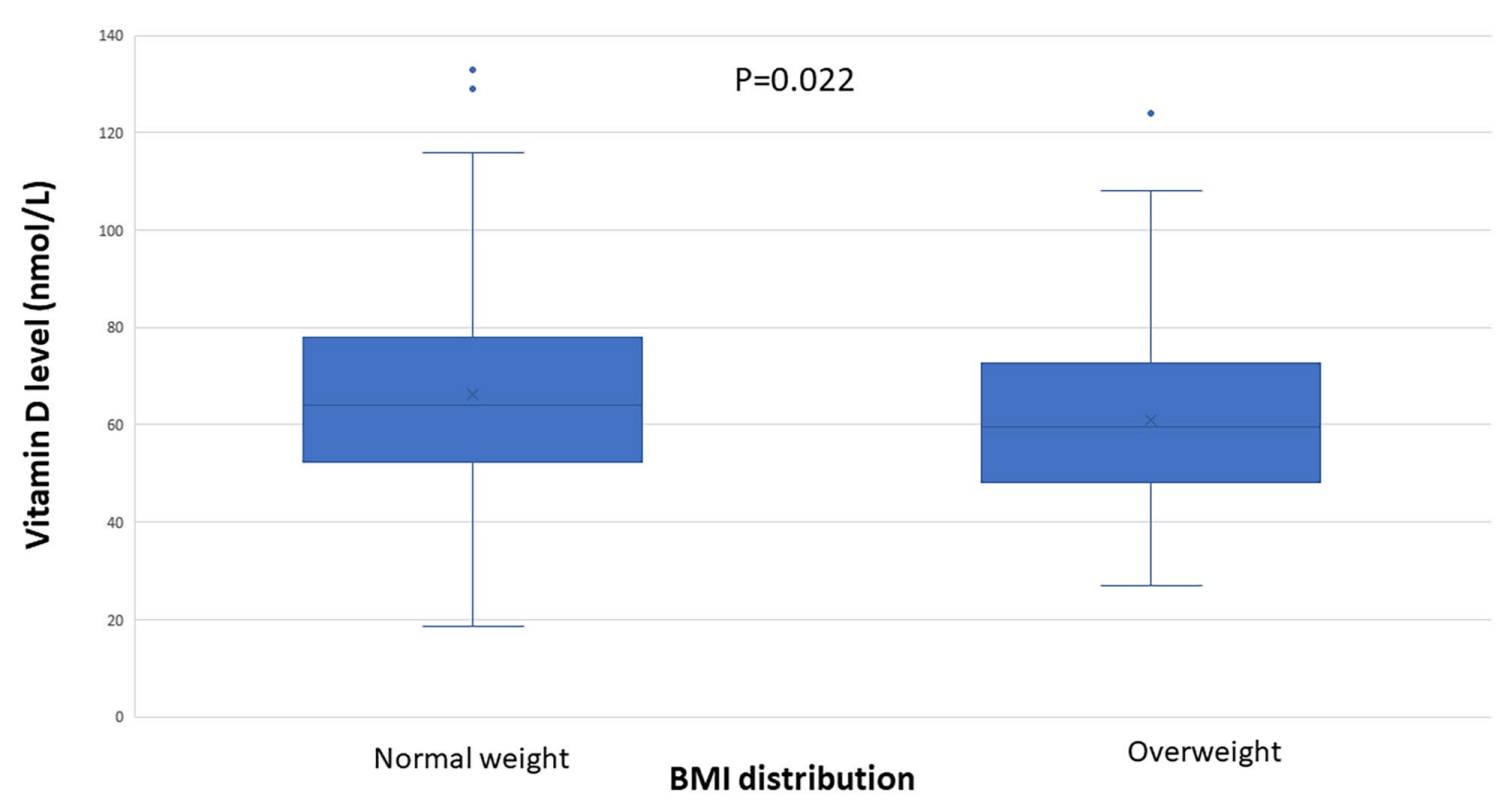
Differences in plasma levels of 25-hydroxy (25-OH) vitamin D depending on normal-weight (body mass index (BMI) < 25 kg/m^2^) or overweight. Mann-Whitney U-test. *P* < 0.05 was considered statistically significant

Most patients and controls had vitamin D levels below reference values (Table 4). Levels of vitamin D < 50 nmol/L, which is the level for recommendation of supplemental treatment [17], was observed in 61 (23.6%) of the IBS patients and in 13 (26.0%) of the controls (*p* = 0.720). IBS patients with overweight had lower vitamin D levels compared with normal weight (60 (48–73) nmol/L vs. 65 (53–78) nmol/L, *p* = 0.022) (Fig. 4), in contrast to equal levels in controls (63 (50–77) nmol/L vs. 63 (49–79) nmol/L). No difference in vitamin D levels was observed between overweight IBS patients and overweight controls (*p* = 0.628). Most of the control samples were collected during the spring whereas the collection among patients were more spread over the year (*p* < 0.001). The vitamin D levels were higher in the autumn than in the spring (*p* < 0.001), but the number of participants with vitamin D levels < 50 nmol/L did not differ between seasons (*p* = 0.122) (Table 5).

**Table 5:** The vitamin D levels stratified for seasonal collection

	Spring *N* = 200	Autumn *N* = 110	*P*-value*	*P*-value**
**25-OH Vitamin D (> 75 nmol/L)** ***Below reference values (n (%))***	158 (79.8)	79 (71.8)		0.122
***Median (interquartile range)***	57 (48–72)	68 (59–80)	< 0.001	
***Season collection*** (n,%)				< 0.001
IBS patients	154 (59.2)	106 (40.8)		
Controls	46 (92.0)	4 (8.0)		

Participants stratified according to blood sampling in the spring or autumn. Mann-Whitney U test* and Fishers exact test** for the number below or above reference values. Values are given as median and interquartile range and number and percentages. *P* < 0.05 was considered statistically significant

Assessment of frequency intake of different food groups showed that the intake of vegetables, dairy products, and cereals was lower in IBS patients than in controls (Table 6). When stratified for the various dietary habits in IBS patients such as gluten reduction (13.8%), lactose free diet (41.9%), and vegetarian diet (9.6%), the only significant finding was a higher level of serum TIBC in the group with vegetarian diet compared with those eating animal products (65 (63–71) µmol/L vs. 61 (55–68) µmol/L, *p* = 0.019). This could not be explained by different ages, since there was no age difference between those who were on vegetarian diet or not (*p* = 0.295).

**Table 6:** The 4-day intake of different food items

	IBS *N* = 132	Controls *N* = 20	*P*-value
**Meat**	4 (2–8)	4 (1.2–7.8)	0.843
**Fish/seafood**	0 (0-1.5)	1 (0–2)	0.174
**Vegetables/legumes**	4 (3–8)	6 (4–8)	0.026
**Fruits/berries**	3 (1–4)	4 (2.2-5)	0.124
**Dairy products**	4 (2–6)	6 (3.2–9.5)	0.004
**Cereals**	8 (4–8)	9 (7.2–11.8)	0.010
**Candies**	4 (2–8)	3 (3–5)	0.891

IBS = irritable bowel syndrome. The total frequency of each item intake for 4 days is given as median and interquartile range of percentages. Mann-Whitney U test. *P* < 0.05 was considered statistically significant

**Fig. 5:**
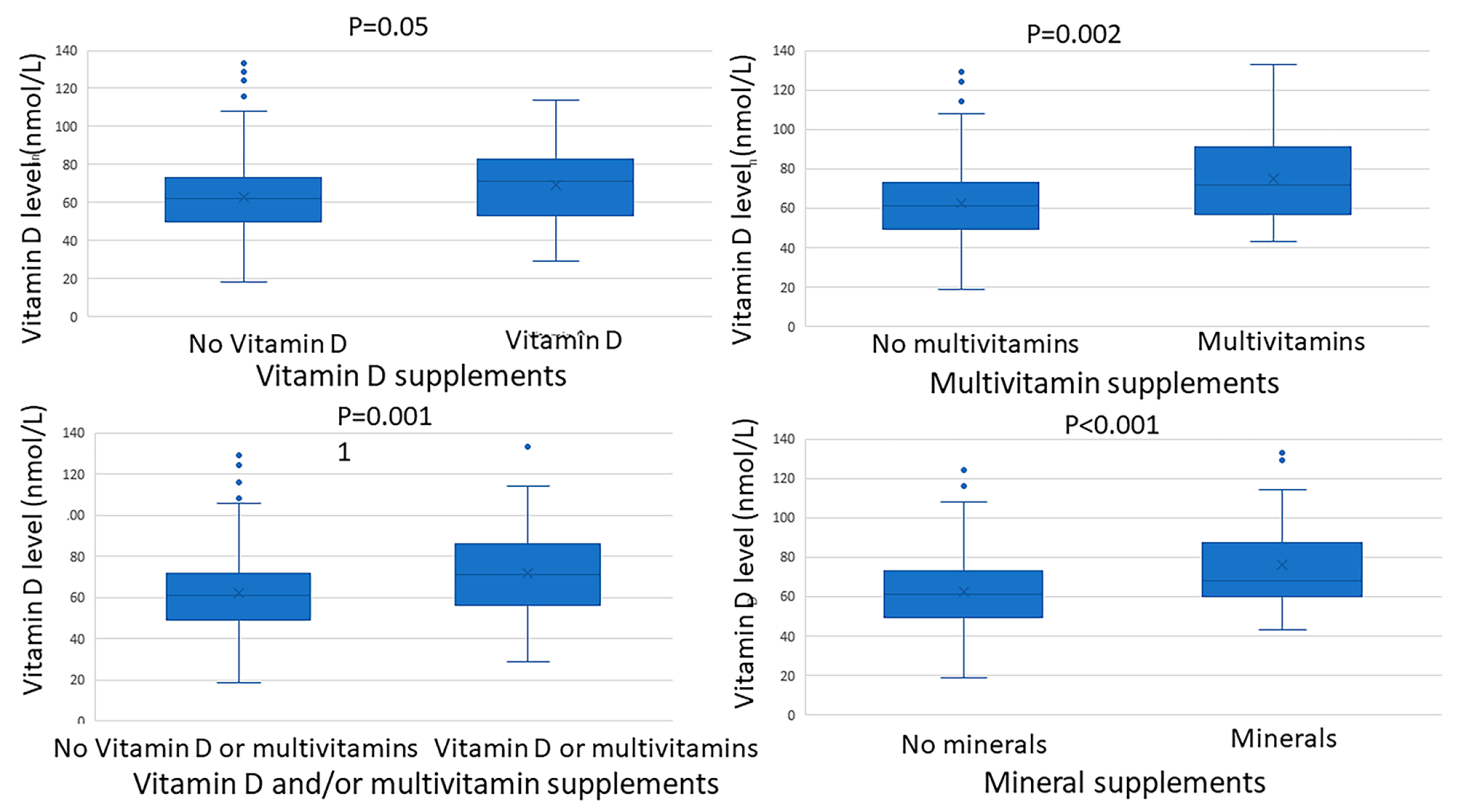
Differences in plasma levels of 25-hydroxy (25-OH) vitamin D depending on intake of vitamin and mineral supplements. Mann-Whitney U-test. *P* < 0.05 was considered statistically significant

When stratified for vitamin and mineral supplements in IBS, higher levels of circulating vitamin D were found in those who used supplements with vitamin D (71 (53–83) nmol/L vs. 62 (50–73) nmol/L, *p* = 0.05), multivitamins (72 (57–91) nmol/L vs. 61 (49–73) nmol/L, *p* = 0.002), vitamin D and/or multivitamins (71 (56–86) vs. 61 (49–72), *p* = 0.001) and minerals (68 (60–87) nmol/L vs. 61 (49–73) nmol/L, *p* < 0.001) than those who did not use nutritional supplements (Fig. 5).

### Correlations

In the whole study cohort, BMI correlated positively with CRP (*p* < 0.001) and ferritin (*p* < 0.001) and correlated inversely with iron (*p* = 0.023), vitamin D (*p* = 0.018), and transferrin saturation (*p* = 0.031). Weight also correlated with CRP (*p* < 0.001) and ferritin (*p* < 0.001) (Table 7). When examining only the patient group, the inverse correlation between BMI and vitamin D was strengthened (*rs*=-0.166, *p* = 0.009).

**Table 7:** Correlations between weight, body mass index (BMI) and laboratory analyses

	Weight	BMI	CRP	Cobalamin	Folate	Ferritin	Iron	TIBC	Transferrin saturation	25-OH Vitamin D
**Weight**			Rs = 0.323, *p* < 0.001			Rs = 0.446, *p* < 0.001				
**BMI**			Rs = 0.434, *p* < 0.001			Rs = 0.382, *p* < 0.001	Rs=-0.127, *p* = 0.027		Rs=-0.125, *p* = 0.031	Rs=-0.142, *p* = 0.014
**CRP**	Rs = 0.323, *p* < 0.001	Rs = 0.434, *p* < 0.001					Rs=-0.277, *p* = 0.001		Rs=-0.277, *p* = 0.001	
**Cobalamin**						Rs = 0.273, *p* < 0.001				Rs = 0.273, *p* < 0.001
**Folate**			Rs=-0.257, *p* = 0.003			Rs=-0.199, *p* = 0.013				Rs = 0.199, *p* = 0.014
**Ferritin**	Rs = 0.446, *p* < 0.001	Rs = 0.382, *p* < 0.001			Rs=-0.199, *p* = 0.013			Rs=-0.432, *p* < 0.001	Rs = 0.240, *p* = 0.003	
**Iron**		Rs=-0.127, *p* = 0.027	Rs=-0.277, *p* = 0.001						Rs = 0.932, *p* < 0.001	
**TIBC**						Rs=-0.432, *p* < 0.001			Rs=-0.412, *p* < 0.001	Rs=-0.171, *p* = 0.003
**Transferrin saturation**		Rs=-0.125, *P* = 0.031	Rs=-0.277, *p* = 0.001			Rs = 0.240, *p* = 0.003	Rs = 0.932, *p* < 0.001	Rs=-0.412, *p* < 0.001		
**25-OH Vitamin D**		Rs=-0.142, *p* = 0.014		Rs = 0.273, *p* < 0.001	Rs = 0.199, *p* = 0.014			Rs=-0.171, *p* = 0.003		

BMI = body mass index, CRP = C-reactive protein, TIBC = total iron-binding capacity, 25-OH = 25-hydroxy. Both IBS patients and healthy controls were included in the calculations with Spearman´s correlation test. *P* < 0.05 was considered statistically significant

CRP correlated inversely with folate (*p* = 0.003), iron (*p* = 0.001), and transferrin saturation (*p* = 0.001). Analyses reflecting the iron content correlated inversely with TIBC. Vitamin D correlated positively with folate (*p* = 0.014) and cobalamin (*p* < 0.001), but inversely with TIBC (*p* = 0.003) (Table 7).

Since age correlated with weight, and age and weight correlated with several micronutrient levels and symptoms, calculations between weight, micronutrient levels, and symptoms in the IBS patients were adjusted for these variables as appropriate. Weight was inversely associated with constipation, but BMI was mostly associated with aggravated extraintestinal symptoms such as headache, back pain, fatigue, leg pain, and muscle/joint pain, which remained statistically significant after FDR adjustment (Table 8). The GI symptom most often associated with micronutrients was bloating and flatulence, which was inversely associated with iron and transferrin saturation, but positively associated with TIBC. The inverse associations between iron and vomiting and nausea and total IBS-SSS were the only significant associations of GI symptoms which remained after FDR. Regarding extraintestinal symptoms, iron was inversely associated with total extraintestinal IBS-SSS (Table 8).

**Table 8:** Associations between circulating micronutrient levels and symptoms in irritable bowel syndrome

	Weight	BMI	Cobalamin	Iron	TIBC	Transferrin saturation
**VAS-IBS**						
**Constipation**	-0.53(-0.80-(-0.26) *p* < 0.001, FDR = 0.019	-1.07(-1.96-(-0.16)) *p* = 0.021, FDR = 0.054	0.03(0.01–0.06) *p* = 0.018,FDR = 0.107			
**Bloating and flatulence**	-0.19(-0.37-0) *p* = 0.050, FDR = 0.150			-0.54(-0.97-(-0.12)) *p* = 0.012, FDR = 0.054	0.26(0.00-0.53) *p* = 0.050, FDR = 0.150	-12.44(-20.78-(-4.11)) *p* = 0.003, FDR = 0.054
**Vomiting and nausea**				-0.967(-1.41-(-0.52)) *p* < 0.001, FDR = 0.018		
**Influence on daily life**				-0.46(-0.85-(-0.04)) *p* = 0.030, FDR = 0.105		
**Psychological well-being**					0.36(0.05–0.66) *p* = 0.022, FDR = 0.108	
***IBS-SSS***						
**Headache**		1.03(0.25–1.80) *p* = 0.010, FDR = 0.030		-0.56(-1.11-(-0.20) *p* = 0.042, FDR = 0.108		
**Back pain**	0.33(0.06–0.59) *p* = 0.016, FDR = 0.072	1.54(0.67–2.41) *p* < 0.001, FDR = 0.009			0.55(0.18–0.92) *p* = 0.004, FDR = 0.072	
**Fatigue**	0.26(0.03–0.49) *p* = 0.029, FDR = 0.104	1.09(0.33–1.84) *p* = 0.005, FDR = 0.018		-0.56(-1.08-(-0.04) *p* = 0.035, FDR = 0.105	0.36(0.04–0.68) *p* = 0.030, FDR = 0.108	
**Reflux**					0.47(0.12–0.83) *p* = 0.009, FDR = 0.081	
**Leg pain**	0.26(0.06–0.46) *p* = 0.009, FDR = 0.072	0.94(0.28–1.59) *p* = 0.005, FDR = 0.018				9.84(0.93–8.76) *p* = 0.030, FDR = 0.213
**Muscle/joint pain**	0.33(0.06–0.59) *p* = 0.015, FDR = 0.072	1.59(0.73–2.44) *p* < 0.001, FDR = 0.009	0.04(0.01–0.07) *p* = 0.007, FDR = 0.063			
**Total IBS-SSS**			0.09 (0.03–0.16) *p* = 0.006, FDR = 0.063	-2.23(-3.65-(-0.81)) *p* = 0.002, FDR = 0.018		
**Total extraintestinal IBS-SSS**		3.20(1.08–5.32) *p* = 0.003, FDR = 0.018		-2.07 (-3.54-(-0.60)) *p* = 0.006, FDR = 0.036	1.05 (0.14–1.96) *p* = 0.024, FDR = 0.108	

Analyses of IBS patients with significant associations. Specific gastrointestinal symptoms were measured by visual analog scale for irritable bowel syndrome (VAS-IBS) [35]. IBS severity scoring system (IBS-SSS) assessed total gastrointestinal and extraintestinal symptoms [28]. Generalized linear model with gastrointestinal symptoms as dependent variable and weight, body mass index (BMI), and micronutrients as predictors, adjusted for age and weight (micronutrients). Values given as β and 95% confidence interval (CI). Crude *p*-values as well as the *p*-values adjusted for false discovery rate (FDR) set at 5% according to the Benjamini-Hochberg method [39] were performed for the calculations. The FDR-adjusted p-values were the main results. *P* < 0.05 was considered statistically significant

## Discussion

The main finding of the present study was that patients with IBS had higher CRP levels and were more often overweight or obese compared with healthy individuals, especially IBS-D and in younger ages. Despite frequent use of vitamin and mineral supplements among IBS patients, and most control samples collected in the spring in contrast to patient samples, levels of iron, vitamin D, and folate were equal or higher in controls than in IBS patients. Vitamin D levels were lower in overweight patients and correlated positively with folate and cobalamin, but inversely with BMI and TIBC, suggesting vitamin D to be a representative biomarker of the general nutritional status. Weight and BMI were inversely associated with constipation, but mainly associated with aggravated extraintestinal symptoms. Few associations were found between specific GI symptoms and micronutrients, but iron was inversely associated with total GI and extraintestinal symptoms.

Although the recruitment process was different in the two cohorts, with more patients from PCC and a third health care center in the first cohort, and more from social media in the latter cohort, the differences between the cohorts were limited. Some of the differences between controls and IBS patients may depend on the slightly higher age in patients, which reflect both the increased degree of education through the years in the population, and occupation with more retired subjects among patients. The difference in prevalence of overweight and obesity was most pronounced in younger age groups, but the difference in the whole cohort remained when adjusted for age. In the overall Swedish population during the same time, 28.3% of people 16–29 years have overweight or obesity, rendering that the prevalence of overweight in the youngest IBS group was 69% higher than in the general population [38]. The prevalence of overweight/obesity is 45.8% in the whole population [38], compared with 48.1% in our total IBS population.

Overweight and obesity are risk factors to develop IBS according to a recent systematic review [40]. In alignment, the prevalence of IBS in obesity is as high as 31% [41, 42]. A correlation between immunological molecules and BMI in IBS patients suggest that the inflammatory factors induced in obesity could contribute to IBS symptoms [43]. The fibromyalgia syndrome (FMS), another chronic pain syndrome, is also associated with overweight and obesity, with more aggravated associated symptoms such as morning stiffness, headache, fatigue, anxiety, depression, and IBS in overweight/obese subjects than in normal weight subjects [44]. Thus, recent research show that obesity is associated with a general enhanced pain sensitivity [44]. Probably due to the overweight, IBS patients have a significantly higher frequency of prediabetes, a risk factor for type 2 diabetes, than healthy controls [45]. The presence of overweight, hyperinsulinemia, and prediabetes may lead to polyneuropathy with ensuing symptoms prior development of overt type 2 diabetes [46, 47]. Another aspect is that IBS-related symptoms, such as increased symptoms after fiber intake, could contribute to difficulties to follow weight-loss programs and a healthy eating pattern, thus resulting in the failure of weight-loss [48].

Vitamin D is mainly synthesized in the skin from 7-dehydroxycholesterol when exposed to ultraviolet (UV) light [24]. Vitamin D receptors are present on almost all human cells [24]. The low levels of vitamin D in the society may depend on less outdoor activities in the modern society with more use of sunscreen and less intake of dairy products. Further, oily fishes for sale are mainly farmed and contain less vitamin D than wild-caught fish [17]. According to a meta-analysis of 24,600 subjects, there is an increased relative risk for association between vitamin D deficiency and obesity [27], in agreement with our findings of lower vitamin D levels in the overweight patients. In a systematic review, most of the included studies showed that vitamin D deficiency increases the risk to develop obesity in adults and elder subjects [26]. The relationship between vitamin D and obesity is bidirectional [49].

Several mechanisms may explain the association between overweight and vitamin D deficiency. Obese patients may have less outdoor activities and more hours in front of computer or television, which reduces the possibility of skin exposure to sunlight and thereby limits the endogenous vitamin D production [50]. Vitamin D is fat-soluble and absorbed by adipose tissue. Vitamin D is released from the adipose tissue at a much slower rate, proportional to the concentration [51]. Vitamin D insufficiency may favor adiposity by increased levels of parathyroid hormones (PTH) and calcium inflow in adipocytes and lead to excessive differentiation between preadipocytes and adipocytes [52]. High expression of vitamin D receptors in the adipose tissue may play a role in the development of the metabolic syndrome [53]. A rat model showed how a vitamin D insufficient diet led to significant increase in body weight compared to a vitamin D adequate diet [54]. The negative effects of vitamin D deficiency on adipose tissue expansion and inflammatory processes with secretion of proinflammatory adipokines along with the development of obesity, suggest a beneficial role of vitamin D on adipocyte metabolism [54]. Hypothetically, vitamin D could be a possible treatment to prevent or reduce obesity.

The elevated CRP levels in the IBS patients are in alignment with importance of inflammation for development of visceral hypersensitivity and IBS [1, 3]. In obese subjects, an association has been described between low vitamin D levels and inflammation [55]. The combination of low vitamin D levels and overweight/obesity in our present cohort may thus have contributed to the development of IBS. Vitamin D deficiency may trigger IBS symptoms [22], through several immunological mechanisms aggravating both peripheral and central pain stimulation leading to chronic pain and discomfort [21, 56]. Randomized controlled trials have shown improvement in IBS symptoms and health-related quality of life after vitamin D supplements [57, 58]. The mechanisms may be modulation of inflammatory mechanisms, since vitamin D associated inversely with BMI and inflammatory biomarkers, whereas BMI associated positively with a variety of inflammatory biomarkers [59]. The effect of vitamin D on other health aspects such as depression and fatigue may be another reason to improved symptoms and quality of life [58]. Furthermore, vitamin D affects mucosal homeostasis, with increased expressions of tight junctions and preserved structural integrity, decreasing the risk for increased permeability and improving the regulation of gut microbiota composition and function [60, 61]. The IBS patients in the current study had lower intake of dairy products but used several vitamin D supplements, which may have modified their experienced symptoms.

We have previously published that several IBS patients have poor dietary habits with high intake of sugar and processed food and low intake of fruits and vegetables [62]. In addition, a high frequency of self-prescribed elimination diets was found, which can further deteriorate micronutrient intake. Vitamin D may be a marker of malnutrition and poor lifestyle habits, since it correlated with BMI, folate, cobalamin, and TIBC. Others have shown that TIBC was the only iron index that was associated with vitamin D deficiency [63]. Thus, vitamin D and TIBC may be the most important analyses in this context. In the current study, TIBC was the only analysis affected in vegetarian diet. The low vitamin D levels also in controls suggest that several in the society have insufficient intake of many micronutrients and/or low number of outdoor activities. Other studies have found lower levels of vitamin D in IBS patients compared with non-IBS patients [64]. Blood samples were mainly collected in the spring in controls and more throughout the year in the patients, which means that controls could have had higher levels compared with IBS if sample collection had been more equal regarding season. Furthermore, the frequent use of vitamin and mineral supplements in the IBS group compensates for the poor nutritional intake by food. If IBS patients had not used so many supplements, they should have had lower values than controls, as observed when comparing vitamin D levels in users or not of vitamin D and minerals. On the other hand, the lower levels of vitamin D in IBS patients may partly be explained by overweight and obesity [26, 27, 48, 50]. PPI was only sparsely used by controls but often used by IBS patients. The risk of malnutrition with lower levels of circulating micronutrients during PPI treatment has been debated for a long time, but no clear conclusions about the PPI effects have been possible to draw in systematic reviews and meta-analyses [65, 66].

There are several micronutrients we never measure, and which are not replaced by vitamin D or other vitamin supplements. Those other micronutrients may also be of great importance for symptom development and the process towards visceral hypersensitivity and chronic pain. Furthermore, environmental exposures, such as air pollutants and microbial exposure, have also been linked to IBS development and gut dysbiosis, and could affect visceral hypersensitivity [67].

This study has several limitations, one being the cross-sectional character of the study, without possibilities to examine causality. At the time point for inclusion, the controls had less weight and BMI, but were also younger. These differences may be confounders in the results described. However, age and weight were adjusted for in the statistical calculations, and participants were divided into age groups for weight comparisons. The question remains whether controls, with their lower vitamin D levels, will develop more overweight and IBS later in life. The small control cohort, with controls from hospital staff and students is a limitation increasing the risk of Type II errors but is explained by the difficulties to recruit controls. However, the use of existing reference values for GI symptoms and blood samples is a strength [36, 37]. The diary books were not completed by all participants, which is a limitation. Furthermore, the registered food items do not necessarily mean the actual intake. Another limitation of the study is that the controls did not provide information about ongoing adherence to varying diets. Therefore, the influence of current diets on laboratory levels could not be examined in the control group. The completion of the symptom questionnaires may be affected by other factors, e.g., psychological factors, and not only the symptoms. However, only validated questionnaires were used, to ensure that the tool reliably and accurately measures what it is intended to measure.

## Conclusions

In conclusion, IBS patients were often overweight or obese with low vitamin D levels. This illustrates the complexity of IBS and the multifactorial nature of its symptoms, including the potential contributions of nutritional deficiencies and comorbid conditions. Further research is needed to explore these associations in greater detail and longitudinal studies must be conducted to establish causative links. Improved awareness of the importance of healthy dietary habits is necessary and treatment of IBS should maybe also include weight-reducing dietary and supplemental treatment of vitamins and mineral deficiencies.

## Data Availability

Data can be delivered by the corresponding author upon request.

## Abbreviations

CRP: C-reactive protein
FODMAP: Fermentable oligosaccharides, disaccharides, monosaccharides, and polyols
IBS: Irritable bowel syndrome
PCC: Primary healthcare centers
SSRD: Starch- and sucrose-reduced diet
TIBC: Total iron-binding capacity
